# Non-viral gene therapy that targets motor neurons *in vivo*

**DOI:** 10.3389/fnmol.2014.00080

**Published:** 2014-10-14

**Authors:** Mary-Louise Rogers, Kevin S. Smith, Dusan Matusica, Matthew Fenech, Lee Hoffman, Robert A. Rush, Nicolas H. Voelcker

**Affiliations:** ^1^Department of Human Physiology, Centre for Neuroscience, Flinders UniversityAdelaide, SA, Australia; ^2^Department of Anatomy and Histology, Centre for Neuroscience, Flinders UniversityAdelaide, SA, Australia; ^3^Department of Chemistry and Biochemistry, South Dakota State UniversityBrookings, SD, USA; ^4^Australian Research Council Centre of Excellence in Convergent Bio-Nano Science and Technology, Mawson Institute, University of South AustraliaAdelaide, SA, Australia

**Keywords:** targeted gene delivery, PEI, PEGylation, retrograde transport, immunogenes, p75NTR

## Abstract

A major challenge in neurological gene therapy is safe delivery of transgenes to sufficient cell numbers from the circulation or periphery. This is particularly difficult for diseases involving spinal cord motor neurons such as amyotrophic lateral sclerosis (ALS). We have examined the feasibility of non-viral gene delivery to spinal motor neurons from intraperitoneal injections of plasmids carried by “immunogene” nanoparticles targeted for axonal retrograde transport using antibodies. PEGylated polyethylenimine (PEI-PEG12) as DNA carrier was conjugated to an antibody (MLR2) to the neurotrophin receptor p75 (p75NTR). We used a plasmid (pVIVO2) designed for *in vivo* gene delivery that produces minimal immune responses, has improved nuclear entry into post mitotic cells and also expresses green fluorescent protein (GFP). MLR2-PEI-PEG12 carried pVIVO2 and was specific for mouse motor neurons in mixed cultures containing astrocytes. While only 8% of motor neurons expressed GFP 72 h post transfection *in vitro,* when the immunogene was given intraperitonealy to neonatal C57BL/6J mice, GFP specific motor neuron expression was observed in 25.4% of lumbar, 18.3% of thoracic and 17.0% of cervical motor neurons, 72 h post transfection. PEI-PEG12 carrying pVIVO2 by itself did not transfect motor neurons *in vivo*, demonstrating the need for specificity via the p75NTR antibody MLR2. This is the first time that specific transfection of spinal motor neurons has been achieved from peripheral delivery of plasmid DNA as part of a non-viral gene delivery agent. These results stress the specificity and feasibility of immunogene delivery targeted for p75NTR expressing motor neurons, but suggests that further improvements are required to increase the transfection efficiency of motor neurons *in vivo*.

## INTRODUCTION

Targeted gene therapy has the potential to be developed for diseases involving death of motor neurons such as amyotrophic lateral sclerosis (ALS). Motor neurons can be transfected by injecting every muscle innervated by spinal motor neurons. Transport of therapy is then by axonal pathways originating from, terminating in, or passing through the injection site. However, this requires many painful injections and even then, it may not be possible to reach all spinal motor neurons (Towne et al., 2011). Alternatively motor neurons can be difficult to access and transfect from the circulation or centrally through injections into the cerebrospinal fluid (CSF). Peripheral injections of viral gene therapy have not been successful at selectively targeting motor neurons (Towne et al., 2008). The blood brain barrier (BBB) is also effective at keeping toxins and infectious material out of the central nervous system (CNS; Pardridge, 2006). Our group has been developing targeted gene delivery agents called “immunogenes” with the aim of using them to deliver therapeutic genes to diseased motor neurons (Rogers and Rush, 2012). They are composed of antibodies that internalize after targeting cell surface receptors and are conjugated to cationic carriers, able to condense DNA/RNA, forming the immunogene. Cells that express the cognate cell-surface receptors of the targeting antibody can therefore be specifically transfected with immunogenes *in vivo* from the circulation (Rogers and Rush, 2012).

Antibodies that internalize into target cells are essential for immunogenes. We previously used an antibody (clone MC192) to the rat common neurotrophin receptor p75 (p75NTR) as a targeting agent (Barati et al., 2006). p75NTR is a receptor highly expressed on motor neurons during the embryonic period, down regulated in adulthood (Yan and Johnson, 1988), only to be re-expressed following neuronal injury, including ALS (Lowry et al., 2001). Past research has revealed that p75NTR is retrogradely trafficked in signaling endosomes in motor neurons when taken up by at distal terminals (Lalli and Schiavo, 2002), rendering this receptor ideally suited to deliver therapeutic genes for motor neurons. Transport from the periphery to motor neurons should be possible using antibodies that target rat p75NTR (Bronfman et al., 2003), i.e., MC192 and pan specific MLR2 (Rogers et al., 2006; Matusica et al., 2008). Both have been demonstrated to internalize with the receptor making them ideal targeting agents.

The development of immunogenes as targeted nanocarriers is particularly attractive for diseases such as ALS. In almost all cases of ALS, death occurs within 3–5 years of diagnosis due to the selective death of motor neurons and there are no effective therapies (Turner et al., 2013). We have previously used immunogenes to deliver therapeutic glial-derived growth factor (GDNF) to injured motor neurons *in vivo* in neonatal rats (Barati et al., 2006). The rat specific p75NTR antibody MC192 was conjugated to a cationic polymer poly(L-lysine; PLL) to condense plasmids expressing GDNF and the immunogene was given intramuscularly (Barati et al., 2006). Although GDNF rescued motor neurons that innervated injected muscles, this first generation immunogene could not be used in the circulation to access larger pools of motor neurons (Barati et al., 2006), making it vulnerable to rapid degradation. Cytotoxicity *in vivo* can be associated with the surface charge of the polymer (Chollet et al., 2002) and poor stability is associated with interactions with erythrocytes and serum components such as albumin, lipoproteins or IgG (Rogers and Rush, 2012). These issues can be overcome by masking the surface charge with agents such as polyethylene glycol (PEG). Forming a hydrophilic shell, PEG limits the hydrophobic or electrostatic interactions with the extracellular medium and prevents binding of the cationic polymer with erythrocytes and plasma proteins (Chollet et al., 2002; Rogers and Rush, 2012). Hence, such measures are required for stealth in the circulation.

After entering cells, non-viral gene delivery agents must be able to escape the endosome/lysosomal compartments to deliver their payload of DNA or RNA to the nucleus and RNA-induced silencing complex (RISC) complex, respectively, (Rogers and Rush, 2012). Our first generation immunogene used PLL that required fusogenic peptides to escape endosomal/lysososomal compartments of cells (Navarro-Quiroga et al., 2002). Other DNA/RNA condensing agents such as polyethylenimine (PEI) have more useful properties including a mechanism for endosomal escape. PEI possesses a high cationic charge density due to secondary amino groups that enables the endosomal/lysosomal release of complexes due to the so-called “proton sponge effect” (Boussif et al., 1995; Tang and Szoka, 1997; Lungwitz et al., 2005). PEI unlike PLL also facilitates the entry of plasmid DNA into the nucleus (Godbey et al., 1999).

Toxicity of intravenously administered cationic polyplexes cannot only be reduced by PEGylation (Merdan et al., 2003; Ogris et al., 2003; Malek et al., 2009) but also when nanoconstructs are also endowed with antibodies or other targeting moieties (Zhang et al., 2003; Luo et al., 2010; Höbel et al., 2011; Schaffert et al., 2011). This may be reflective of specificity in addition to lower toxicity because of reduction in charge after conjugation to for example an antibody. Besides systemic toxicities, cytotoxic effects are also observed upon polyplex internalization. Since polycations electrostatically bind and condense DNA, non-specific electrostatic binding to any kind of cellular polyanions (e.g., enzymes, mRNA, or genomic DNA) may deregulate the expression profile of housekeeping genes (Godbey et al., 2001) or induce activation of genes involved in apoptosis (Masago et al., 2007). Consequently, characteristics of cationic polyplex formulations such as molecular weight, cationic charge density and the presence of free polymer also influence their cytotoxicity (Kunath et al., 2003; Boeckle et al., 2004; van Gaal et al., 2011). Accordingly, we hypothesize that an ideal candidate for a safe non-viral gene delivery vector is a carrier with a neutral to slightly negative charge and the capability of being targeted to the cell type required.

In addition to targeting cells from the periphery, we also aimed to improve the expression of transgenes. Methods to improve nuclear import of plasmids are of particular importance in post mitotic cells such as motor neurons. Transfection rates can be poor in post mitotic cells as there is limited breakdown of the nuclear envelope (Zabner et al., 1995). Therefore, modifying plasmids to improve nuclear entry is required. One way of achieving this is to include in the plasmid design a DNA targeting sequence (DTS) that bind to endogenously expressed transcription factors that then act as nuclear localization sequences (NLSs) and improve nuclear import (Mandke and Singh, 2012). Plasmid vectors also often contain sites that can produce innate immune responses through unmethylated cytosine guanine bases separated by only one phosphate (CpGs; Magnusson et al., 2011). Removal of CpGs from the plasmid backbone has been shown to reduce immune reactions to plasmids and prolong expression *in vivo* (Magnusson et al., 2011; Davies et al., 2012). Hence, plasmids that are chosen for *in vivo* delivery should include DTS and minimal CpGs.

Here, we report on the development and evaluation of immunogenes capable of targeting motor neurons *in vitro* and *in vivo*. We demonstrate specificity of delivery to motor neurons can be achieved from peripheral injections using p75NTR antibody MLR2. We show that nanocarriers comprised of p75NTR antibody MLR2 conjugated to PEGylated PEI can deliver plasmids to mouse motor neurons *in vitro* and *in vivo*. In addition, we demonstrate gene expression in motor neurons *in vivo* using plasmids designed for improved nuclear entry and less immunogenicity. Hence, we explore the potential of using p75NTR-targeting immunogenes as gene therapy.

## MATERIALS AND METHODS

### PREPARATION OF NANOCONSTRUCTS

**FIGURE 1:**
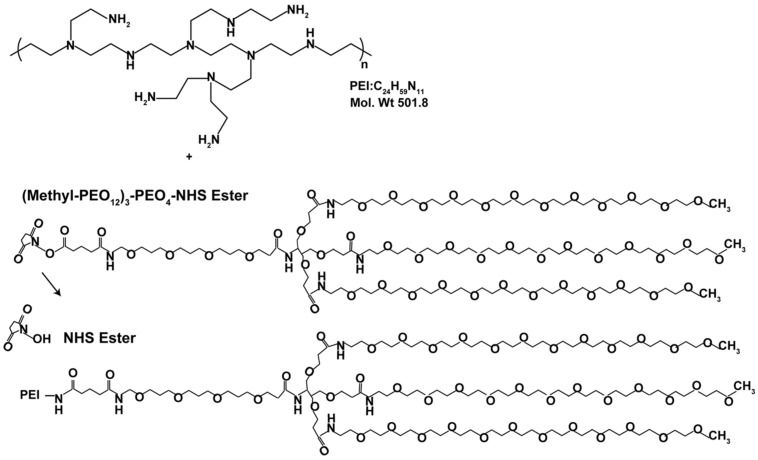
**Stepwise synthesis of PEGylated PEI**.

Branched PEI (C_24_H_59_N_11_PEI, molecular weight 25 kDa; Sigma Aldrich, Australia) was made to 20 mg/ml in H_2_O and deprotonated with HCl to pH 7.0. PEI was then buffer exchanged on PD10 columns (GE, Australia) with 20 mM 4-(2-hydroxyethyl)-1-piperazine ethane sulfonic acid (HEPES; Invitrogen, Aust), 250 mM NaCl, and pH 7.9. 50 mg of PEI was PEGylated with a branched PEG reagent (Methyl-PEO_12_)_3_-PEO_4_-NHS ester (Thermo Scientific, Rockford, IL, USA) with a molecular weight of 2421 g/mol, (**Figure 1**). This was achieved at a molar ratio of 10:1 PEG to PEI. The number of PEGs per PEI was analyzed by spectral analysis using a Varian 300 MHz NMR spectrometer NMR with deuterium oxide (D_2_O) as the solvent indicating on average 12 PEG moieties conjugated per PEI, corresponding to 6% of amines on the PEI being PEGylated. Hybridoma MLR2 was grown and MLR2 purified on protein G column as previously described (Rogers et al., 2006). Conjugation of PEI-PEG12 or PEI to anti-p75NTR MLR2 was achieved using methods adapted from Blessing et al. (2001) and Germershaus et al. (2006). Briefly, the cross-linker *N*-succinimidyl 3-(2-pyridyldithio)propionate (SPDP; Sigma Aldrich, Australia) was used to produce SPDP-activated PEI-PEG12, PEI, and MLR2 IgG. A molar ratio of 1.62 SPDP to PEI was found to generate one functional SPDP-activated PEI-PEG12. PEI without PEG12 was also functionalized using the same method but at a molar ratio of 1.3 SPDP to PEI to generate one SPDP activated PEI. Twenty molar excess DTT was used to generate a thiol-functionalized PEI-PEG12 and PEI. The reactions were all conducted for 1 h. MLR2 IgG was activated with SPDP (at a molar ratio of 4 SPDP: 1 IgG for 2 h), to produce 1 functional SPDP-activated MLR2. To produce the conjugate, thiol-functionalized PEI-PEG12 or PEI was mixed with SPDP-activated MLR2 at a molar ratio of 2:1, and reacted for 24 h under nitrogen gas atmosphere. The amount of PEI-PEG12 or PEI per IgG was calculated as 1.3 to 1 after measuring the release of pyridine-2-thione (343 nm). All reactions were in a reaction buffer of 20 mM HEPES, 250 mM NaCl, pH 7.9, and after each step constructs were purified by gel filtration on PD-10 columns. PEI concentration was calculated by TNBS (2,4,6-trinitrobenzene sulfonic acid) assay using a standard PEI dilution curve as previously described (Snyder and Sobocinski, 1975). The conjugate was purified using cation exchange on HiTrap SP Sepharose (GE, Australia) with stepwise NaCl elution of 1.0, 2.0, and 3.0 M NaCl in 20 mM HEPES pH 7.2. The MLR2-PEI-PEG12 conjugate was eluted with 2.0 M NaCl, and the construct MLR2-PEI with 3.0 M NaCl. A 100 kDa cut-off Ultra 4 (Millipore) centrifuge column was used to replace the high salt with isotonic buffer (20 mM HEPES, 0.15 M NaCl, pH 7.3.

### PREPARATION OF PLASMID DNA

An enhanced green fluorescent protein (GFP) expressing plasmid was from Aldevron (pgWiZ; Fargo, ND, USA). This plasmid was used for some *in vitro* transfection experiments and although produces sustained GFP expression it can induce immune responses *in vivo* (Chamarthy et al., 2003; Grønevik et al., 2005; Rose et al., 2014). Hence for *in vivo* work a bicistronic pVIVO2 plasmid (9.6 kb) was purchased from Invivogen (San Diego CA, USA). pVIVO2 includes a SV40 DNA targeting signal (DTS) for improved nuclear entry with cytosine and guanine separated by only one phosphate (CpG) motifs removed from the plasmid backbone to reduce immune reactions *in vivo* (Davies et al., 2012). pVIVO2 also contains two human ferritin composite promoters, FerH (heavy chain) and FerL (light chain) combined with SV40 and CMV enhancers for GFP and LacZ expression, respectively. Competent *Escherichia coli* cells were transformed with pgWiZ or pVIVO2 plasmids and purified using Endotoxin Free Maxi Prep Kits (Qiagen) as per the manufacturer’s instructions.

### SIZE AND ZETA POTENTIAL AND GEL RETARDATION

Nanoconstructs were subject to size (nm) and charge measurements (zeta potential in mV) using a Malvern Zetasizer Nano. Zeta potential is a measure of the magnitude of particle charge in solution. Briefly, MLR2-PEI-PEG12 or PEI-PEG12 was mixed with plasmid (pgWiZ) at a nitrogen (amine) to phosphate (DNA) ratio (N/P) of 2, 5, 10, and 12 in sample buffer (20 mM HEPES, 0.15 M NaCl, pH 7.3). Samples were placed in a disposable capillary cell (DTS 1060) where both zeta potential and particle size were measured. The charge at each N/P ratio was analyzed in gel-retardation assays as described (Kircheis et al., 1997). Briefly, samples (10 μl) containing 400 ng pDNA and varying amount of conjugate at different N/P ratios were applied to 1% agarose gels made in Tris-Borate-EDTA buffer with GelRed^TM^ at 1/10,000 (Biotium, Hayward, CA, US) at 100 V for 60 min. The gel was then imaged on a using BioRad Gel Doc 2000 transilluminator (Bio-Rad Laboratories, Hercules, CA, USA).

### CELL CULTURE AND CYTOTOXICITY ASSAYS

Primary motor neurons (PMN) were isolated from E12.5 embryonic mouse (C57BL/6J) spinal cords as previously described (Wiese et al., 2010) or as mixed motor neuron/glia cultures (Ford et al., 1994) and cells cultured on 48-well plates (Nunc) coated with poly-D-ornithine/laminin (Wiese et al., 2010). Motor neurons were grown in Neurobasal media (Invitrogen) supplemented with 10% horse serum, GlutaMAX, B27 supplement (Invitrogen) and 10 nM β-mercaptoethanol and BDNF and CNTF (10 ng/ml; Invitrogen, Aust) as previously described (Wiese et al., 2010). Plasmids used for transfection were pgWiZ or pVIVO2 (both expressing GFP). Motor neurons were transfected in cell culture media (without horse serum or β-mercaptoethanol) for 4 h using the polyplexes MLR2-PEI, MLR2-PEI-PEG12, PEI-PEG12, and 20 μg of plasmid (pGwiZ or pVIVO2). Transfectants were removed after 4 h before replacing with full culture media. Viable motor neurons were examined before and after transfection for a total of 7 days in five separate wells using a Leica IX70 inverted fluorescence microscope. Transfection was measured by counting motor neurons expressing GFP detected by microscopy as a percentage of motor neurons plated in at least five wells. Mouse NSC34 motor neuron-like cells, human SHSY5Y and fibroblast control cells were cultured as previously described (Rogers et al., 2010; Shepheard et al., 2014). Flow cytometry for determining labeled antibody specificity is exactly as described previously (Rogers et al., 2010) using an Accuri C6 Flow Cytometry (BD).

### ANTIBODY AND GENE DELIVERY IN C57BL/6J MICE

Approval to undertake experiments using C57BL/6J mice described in this current study was by the Flinders University Animal Welfare Committee. Antibody to p75NTR (MLR2) was labeled with 4 fluorescent dye molecules (Atto-488-NHS-Ester; Sigma) per antibody molecule, as described by the manufacturer. The degree of labeling (DOL) was determined by absorbance of labeled antibody at 501 and 280 nm with the appropriate extinction coefficients and corrections for DOL. Intraperitoneal injections of labeled antibody or immunogenes were given to newborn C57BL/6J neonatal mice, always in two equal doses. After 3–4 days, mice were euthanized and transcardially perfused with PBS containing 1% sodium nitrite, followed by Zamboni’s fixative (4% paraformaldehyde (w/v), 7.5% saturated picric acid (v/v), PBS, pH 7.4). Spinal cords and dorsal root ganglia (DRG) were removed and post fixed overnight in Zamboni’s fixative at 4°C and then cryoprotected in PBS containing 30% sucrose (w/v). 30 and 10 μm sections were cut from spinal cords and DRGs embedded in OCT on a cryostat. Sections were blocked in blocking diluent (PBS with 10% donkey serum (Sigma-Aldrich), 0.2% Tween-20, 0.02% azide) and antibodies incubated in antibody diluent (PBS with 1% donkey serum (Sigma-Aldrich), 0.2% Tween-20, 0.02% azide). Primary antibodies used were rabbit anti homeobox transcription factor 9 (Hb9 used at 1:1000; Abcam, unavailable post 2012); rabbit anti-Choline Acetyltransferase (ChAT) P3YEB (a generous gift from Prof Dr. M. Schemann, Techn Univ Munich, 1:5000), and goat anti-mouse p75NTR (Sigma; 1 μg/ml) and chicken anti-GFP (Biosensis; 1/500). Secondary antibodies included donkey anti sheep-488, donkey anti rabbit-CY3, and donkey anti-chicken-488 (Jackson ImmunoResearch Laboratories). All secondary antibodies were diluted to 1:800. Imaging was carried out on an Olympus BX50 fluorescence microscope.

## RESULTS

### CONSTRUCTION AND CHEMICAL PROPERTIES OF NANOCONSTRUCTS

**FIGURE 2:**
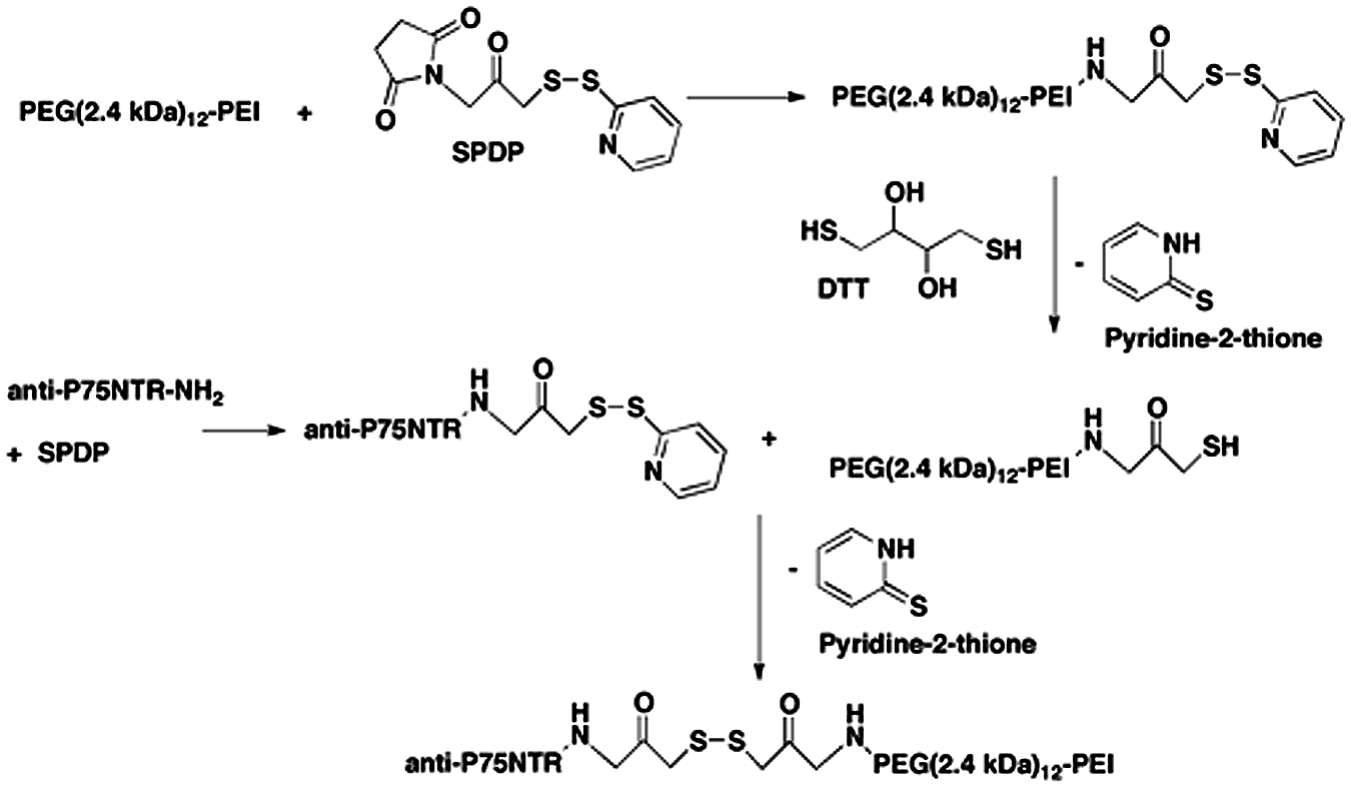
**Stepwise synthesis of the targeted nanoconstructs**.

Branched PEI was used as a DNA condensing agent in the nanoconstructs. Each PEI molecule was PEGylated with 12 PEG moieties, each being 2.4 kDA in molecular weight (**Figure 1**). To engineer specificity of nanoconstructs for motor neurons expressing the cell surface receptor p75 neurotrophin receptor (p75NTR), PEI-PEG12 was conjugated to a monoclonal antibody p75NTR (MLR2; Rogers et al., 2006) using methods adapted from Blessing et al. (2001) and Germershaus et al. (2006) and shown in **Figure 2**. The final construct contains a disulfide bond between an amine on the antibody and an amine on the PEI.

**FIGURE 3:**
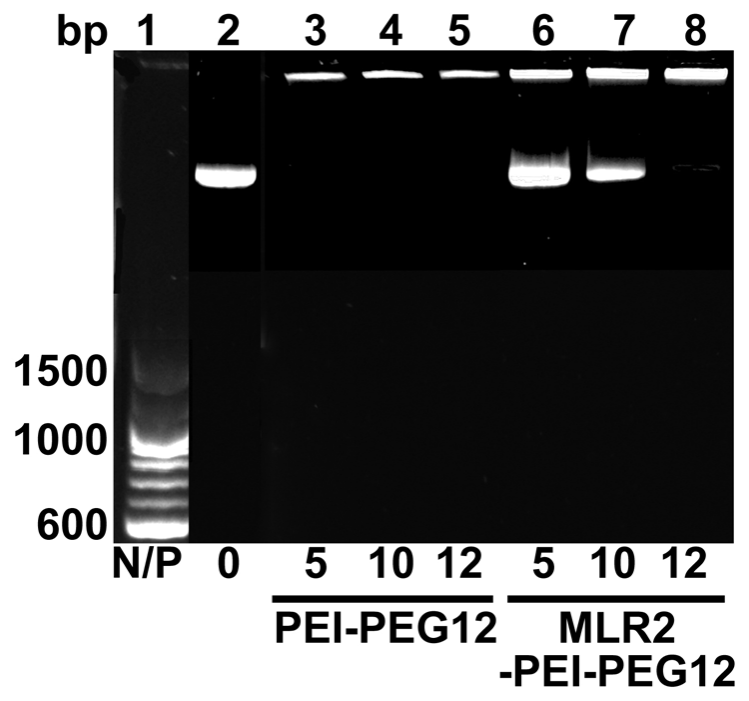
**Agarose gel retardation assay of MLR2-PEI-PEG12-pVIVO2 and PEI-PEG12-pVIVO2.** Lane 1, 100 bp ladder; lane 2, naked pVIVO2 (400 ng); lane 3–5 400 ng pVIVO2 with PEI-PEG12 at N/P 5, 10, and 12; lanes 6–8 400 ng pVIVO2 with MLR2-PEI-PEG12 at N/P 5, 10, and 12.

Gel retardation was used to monitor electrostatic interactions between cationic amines (Nitrogen) in the PEI and the anionic phosphate group of the plasmid DNA (pgWiZ or pVIVO2). This procedure showed the PEI (N): plasmid (P) DNA ratio required to generate a neutral complex. **Figure 3** shows that an N/P of 10 (lane 7) and 12 (lane 8) retarded the complex MLR2-PEI-PEG12-pVIVO2 in the loading well. This is in contrast to PEI-PEG12-pVIVO2 where the complex was retarded with a N/P of 5 (lane 3), indicating that the full immunogene had a less positive charge than PEGylated PEI lacking the antibody. Exactly the same results were obtained if pVIVO2 was replaced with pgWiZ. The charge of the immunogene was confirmed by measuring zeta potential. **Table 1** shows that MLR2-PEI-PEG12 complexed to pgWiZ at N/P 12 had a zeta potential of -19.91 ± 1 mV, in contrast to PEI-PEG12 complexed to pgWiZ with a zeta potential of 4.8 ± 0.9 mV at N/P 12. The size of the MLR2-PEI-PEG12 complexed to plasmid at N/P 12 was 95.3 ± 11 nm, indicating that the DNA was condensed. PEI-PEG12 was 101.1 ± 16.1 nm in size at N/P 12.

**Table 1:** Size and zeta potential of nanoconstructs.

Complex	N/P Ratio	Zeta potential (mV)	Particle size (nm)
PEI-PEG12	2	-14.4 ± 4.7	88.7 ± 13.2
PEI-PEG12	5	-0.5 ± 2.9	78.0 ± 15
PEI-PEG12	10	0.7 ± 3.1	75.8 ± 14
PEI-PEG12	12	4.8 ± 0.9	101.1 ± 16.1
MLR2-PEI-PEG12	5	-42.0 ± 0.4	78.3 ± 14
MLR2-PEI-PEG12	10	-32.5 ± 1.3	82.7 ± 10
MLR2-PEI-PEG12	12	-19.9 ± 1.3	95.3 ± 11

### CYTOTOXICITY AND *IN VITRO* SPECIFICITY OF NANOCONSTRUCTS

**FIGURE 4:**
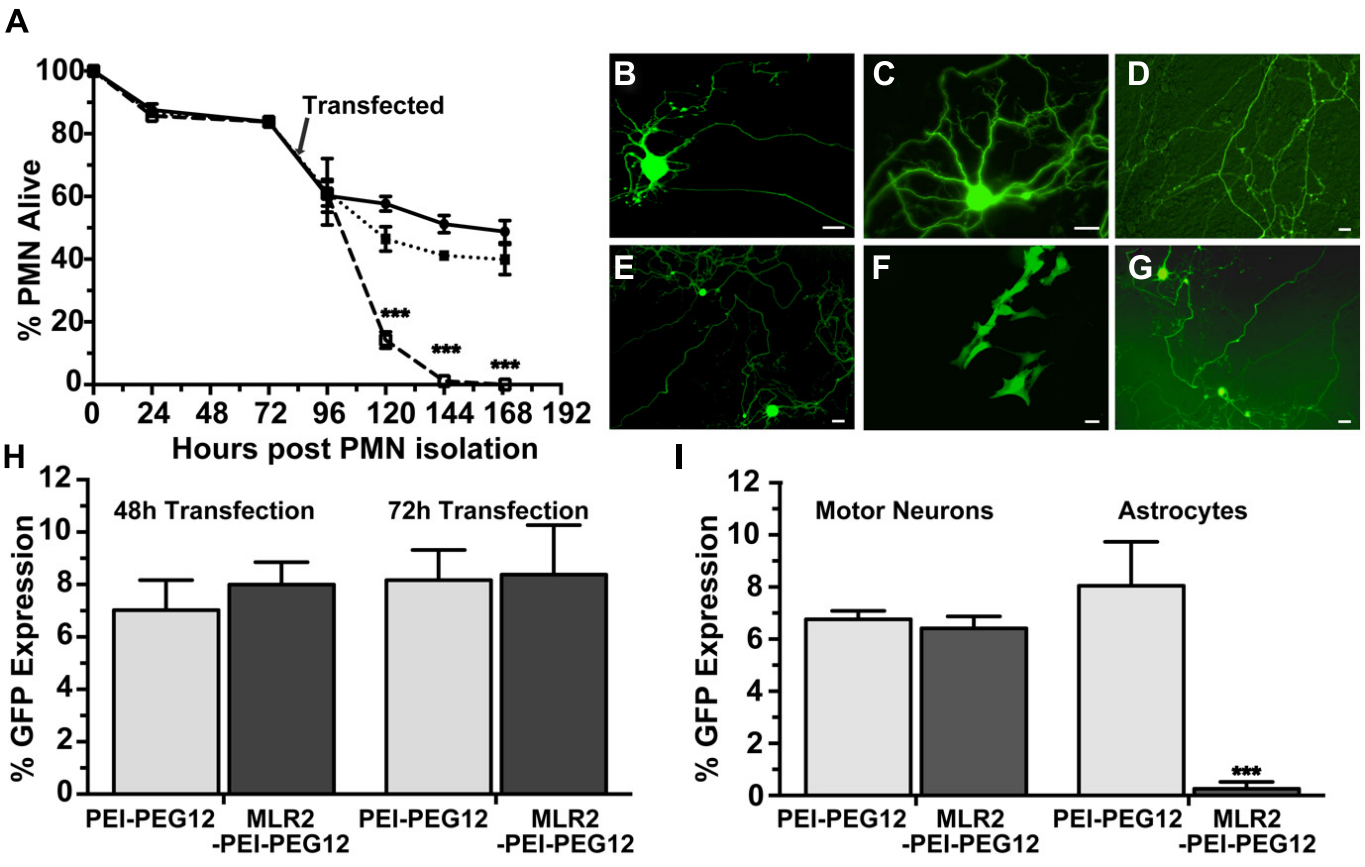
**Transfection of mouse primary motor neurons (PMN) with plasmid DNA using targeted nanoconstructs. (A)** PMN were isolated from E12.5 embryos and transfected 3 days later with pGwiZ carried by MLR2-PEI (open squares) or MLR2-PEI-PEG12 (closed squares). The number of alive and dead motor neurons for each treatment or no treatment (closed circles) were counted every 24 h and the % of neurons that were alive calculated from the original 3000 neurons plated per well in 48-well plates (*n* = 5). There was significantly (^∗∗∗^*p* < 0.001) less PMN alive after treatment with MLR2-PEI-pGwiZ. **(B)** Green fluorescent protein (GFP) expression in live pure motor neurons 48 h after transfection with by MLR2-PEI-PEG12-pVIVO2. **(C,D)** GFP expression after transfection with MLR2-PEI-PEG12-pGwiZ. **(E,G)** GFP expression in motor neurons or **(F)** Astrocytes after transfection with PEI-PEG12-pVIVO2. (scale bar: 20 μm). **(H)** The % of original (3000) motor neurons expressing GFP was determined 48 and 72 h post transfection of pure motor neurons with MLR2-PEI-PEG12-pGwiZ and PEI-PEG12-pGwiZ. **(I)**. Mixed cultures of motor neurons and astrocytes were isolated from embryonic mice spinal cords and 3000 cells plated per well in 48-well plates. The % of transfected motor neurons and astrocytes were determined 72 h after transfection with MLR2-PEI-PEG12-pGwiZ and PEI-PEG12-pGwiZ. There was significantly (^∗∗∗^*p* < 0.001) less astrocyte transfection with MLR2-PEI-PEG12-pGwiZ, compared to PEI-PEG12-pGwiZ.

We next examined the cytotoxicity and transfection ability of immunogenes for motor neurons *in vitro*. PMN were isolated from embryonic mice as previously described (Wiese et al., 2010) in 48-well plates and 4 days later transfected with plasmids (pgWiZ or pVIVO2) expressing GFP, using MLR2-PEI or MLR2-PEI-PEG12. We counted viable motor neurons before and after transfection (**Figure 4A**) for a total of 7 days (*n* = 3 motor neuron isolations in five separate wells). The viability of cells transfected with MLR2-PEI-PEG12-pgWiz was not significantly different than for non-transfected cells over this time period. 48 h and 72 h post transfection with MLR2-PEI-PEG12-pGwiZ there were 46.4 ± 3.5 and 41.1 ± 0.7% of the original viable motor neurons present. This was not significantly different from control non-transfected cells where there were 57.7 ± 2.4 and 51.2 ± 2.7% of original viable motor neurons present at that same time period. However, when PEI was not PEGylated, the number of live motor neurons was significantly (*p* < 0.001) reduced to 14.2 ± 2.6% then 1.1 ± 0.35%, 48 and 72 h post transfection with MLR2-PEI-pGwiZ (**Figure 4A**). There was no significant difference in the percentage of live motor neurons if pVIVO2 was used in place of pgWiZ (results not shown).

GFP expression in pure motor neurons 48 h after transfection is demonstrated with pVIVO2 (**Figure 4B**) or pgWiZ (**Figures 4C,D**) carried by MLR2-PEI-PEG12. **Figure 4C** shows GFP expression in the cell body and processes of a motor neuron and **Figure 4D** shows GFP-containing transfected neuronal processes over a bed of non-transfected cells. GFP expression was also observed in motor neurons after transfection with pVIVO2 carried by PEI-PEG12 (**Figures 4E,G**), and again there is cell bodies and processes with GFP and also non-transfected cells. The percentage of motor neurons expressing GFP was determined 48 and 72 h post transfection with MLR2-PEI-PEG12-pgWiz and PEI-PEG12-pgWiz and MLR2-PEI-pgWiz (**Figure 4H**). Notably, MLR2-PEI-pgWiz did not produce any GFP possibly because few live motor neurons were present after 48 h. However, 8.0 ± 0.8% of motor neurons expressed GFP 48 h after transfection by MLR2-PEI-PEG12-pgWiz and this did not increase significantly by 72 h (8.3 ± 1.8%). Similarly, 7.0 ± 1.15% of motor neurons expressed GFP 48 h post transfection with PEI-PEG12-pgWiz and this rose to 8.1 ± 1.5% by 72 h post transfection (**Figure 4H**).

Mixed cultures of motor neurons and astrocytes were isolated from embryonic mice spinal cords (Ford et al., 1994) and transfected with MLR2-PEI-PEG12-pgWiz or PEI-PEG12-pgWiz. The percentage of motor neurons transfected after 48 h was 6.7 ± 0.32% for MLR2-PEI-PEG12-pGWIZ and 8.0 ± 1.6% for PEI-PEG12-pgWiz. However, 8.0 ± 1.6% of astrocytes were transfected with PEI-PEG12-pgWiz and significantly (*p* < 0.001) less (0.3 ± 0.3%) with MLR2-PEI-PEG12-pgWiz (**Figure 4I**). This demonstrates the selectivity of MLR2-PEI-PEG12 for motor neurons. **Figure 4F** shows GFP expression in astrocytes 48 h post transfection with PEI-PEG12-pgWiz.

### SPECIFICITY OF ANTI-p75NTR (MLR2) AND RETROGRADE TRANSPORT *IN VIVO*

**FIGURE 5:**
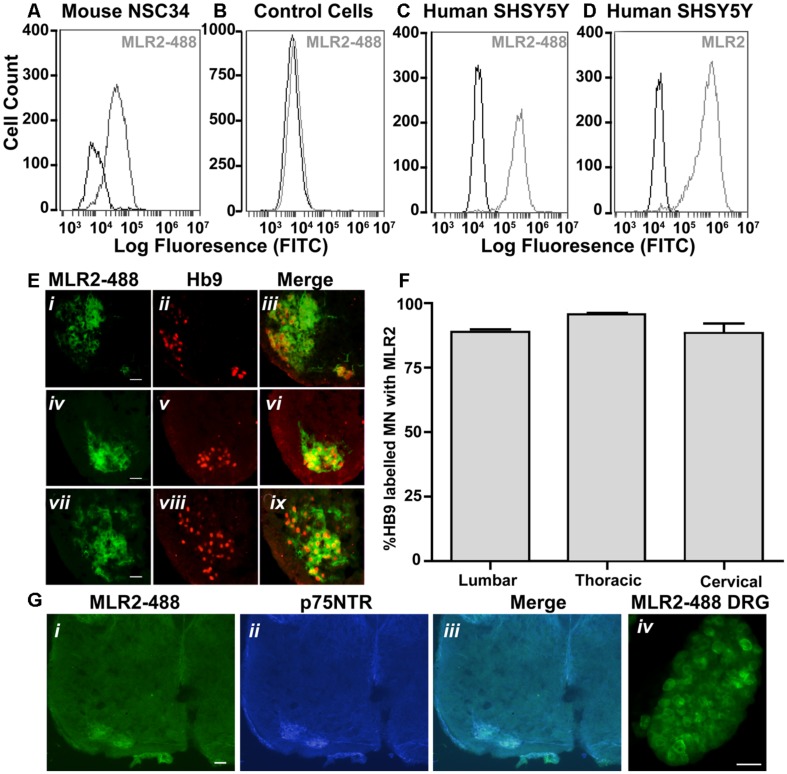
**MLR2 is specific for p75NTR and is transported to the majority of spinal motor neurons after intraperitoneal delivery into neonatal mice **(A–D)** Flow cytometry histograms demonstrating specificity of MLR2-488.** Mouse motor neuron-like cells **(A)**, control fibroblasts **(B)**, human SHSY5Y neuroblastoma cells **(C)** were incubated with and without 20 μg of fluorescently labeled anti-p75NTR (MLR2-488) and fluorescence measured by flow cytometry. X-axis is FITC fluorescence intensity; Y-axis is number of cells displaying FITC fluorescence. Flow cytometry histogram from human SHSY5Y **(D)** incubated with 20 mg unlabeled MLR2 and then anti-mouse 488 (1/100) is included as control for MLR2. **(E)** 488-Fluoresecence observed in lumbar (i), thoracic (iv), and cervical (vii) regions of spinal cord sections of neonatal C57BL/6J spinal cord 36 h after two intraperitoneal injections of 75 μg of MLR2-488; scale bar: 90 μm. Motor neurons identified by anti-Hb9 (1/1000; ii,v,viii); also contained MLR2-488 (iii,vi,xi). **(F)** The majority of Hb9 labeled motor neurons also contained MLR2-488. The % of motor neurons containing MLR2-488 was calculated by counting neurons with Hb9 staining and neurons with MLR2-488 for 30 μm sections from the lumbar, thoracic and cervical regions (*n* = 3 mice with SEM). Motor neurons with MLR2-488-Fluoresecence also contained p75NTR (**G** i,ii,iii; scale bar 50 μm). MLR2-488 was also found in Dorsal root ganglion (from L2-L4; scale bar 80 μm) after intraperitoneal injections of MLR2-488.

A key requirement for *in vivo* gene therapy is specificity to the target cell population. We used an antibody to p75NTR to target motor neurons and sought to demonstrate specificity and usefulness in neonatal mice where high numbers of motor neurons that express p75NTR occur. MLR2 was fluorescently labeled with Atto-488) and the specificity of the labeled antibody for p75NTR determined by flow cytometry. Cells expressing mouse p75NTR (**Figure 5A**) and human p75NTR (**Figure 5C**) were incubated with and without 20 μg/ml labeled MLR2 and subjected to flow cytometry analysis. The shift in mean fluorescence intensity to the right indicates an increase in the antibody binding to the cells. However, there was no change in fluorescence intensity after control fibroblasts lacking p75NTR were incubated with 20 μg/ml labeled antibody (**Figure 5B**), indicating that the antibody indeed specifically targets p75NTR-expressing cells. Unlabeled MLR2 (with secondary anti-mouse antibody labeled with Alexia-Fluor-488) bound to human SHSY5Y cells as expected (**Figure 5D**). Unlabeled MLR2 was not tested on mouse NSC34 cells because the secondary antibody binds non-specifically to mouse derived cells.

Having demonstrated the specificity of the MLR2 antibody for p75NTR, we next tested the ability of MLR2 to be retrogradely transported to spinal cord motor neurons in neonatal mice from the circulation. Two doses of 75 μg of Atto-488 labeled MLR2 (150 μg total) were injected into neonatal B6 mice (average weight was 2 g; *n* = 3) and 36 h later, mice were perfused and spinal cords excised. Lumbar, thoracic and cervical sections were examined for motor neuron marker homeobox transcription factor 9 (Hb9; Red; nuclear stain) and labeled MLR2 (green). Representative micrographs show MLR2 and Hb9 in lumbar (**Figure 5E** i,ii), thoracic (**Figure 5E** iv,v) and cervical (**Figure 5E** vii,viii) sections. Merged images (**Figure 5E** iii,vi,ix) show that the majority of motor neurons identified by Hb9 also contained MLR2. The extent of retrograde transport was assessed for lumbar, thoracic and cervical regions by counting the number of motor neurons labeled with Hb9 and MLR2 and with both labels. **Figure 5F** shows pooled results from three mice; 88.6 ± 1.0% lumbar, 95.7 ± 0.5% thoracic and 87.3 ± 3.8% of cervical motor neurons identified by Hb9 label contained MLR2. Hence, MLR2 is efficiently transported to motor neurons from the circulation in neonatal mice. As expected the motor neurons from mice injected with MLR2-488 also contained p75NTR (**Figure 5G** i,ii, and iii) and MLR2-488 was also found in the p75NTR-expressing neurons of the dorsal root ganglia (DRGs; **Figure 5G** iv).

### RETROGRADE TRANSPORT AND DELIVERY OF MLR2-PEI-PEG12-PVIVO2 TO MOTOR NEURONS *IN VIVO*

**FIGURE 6:**
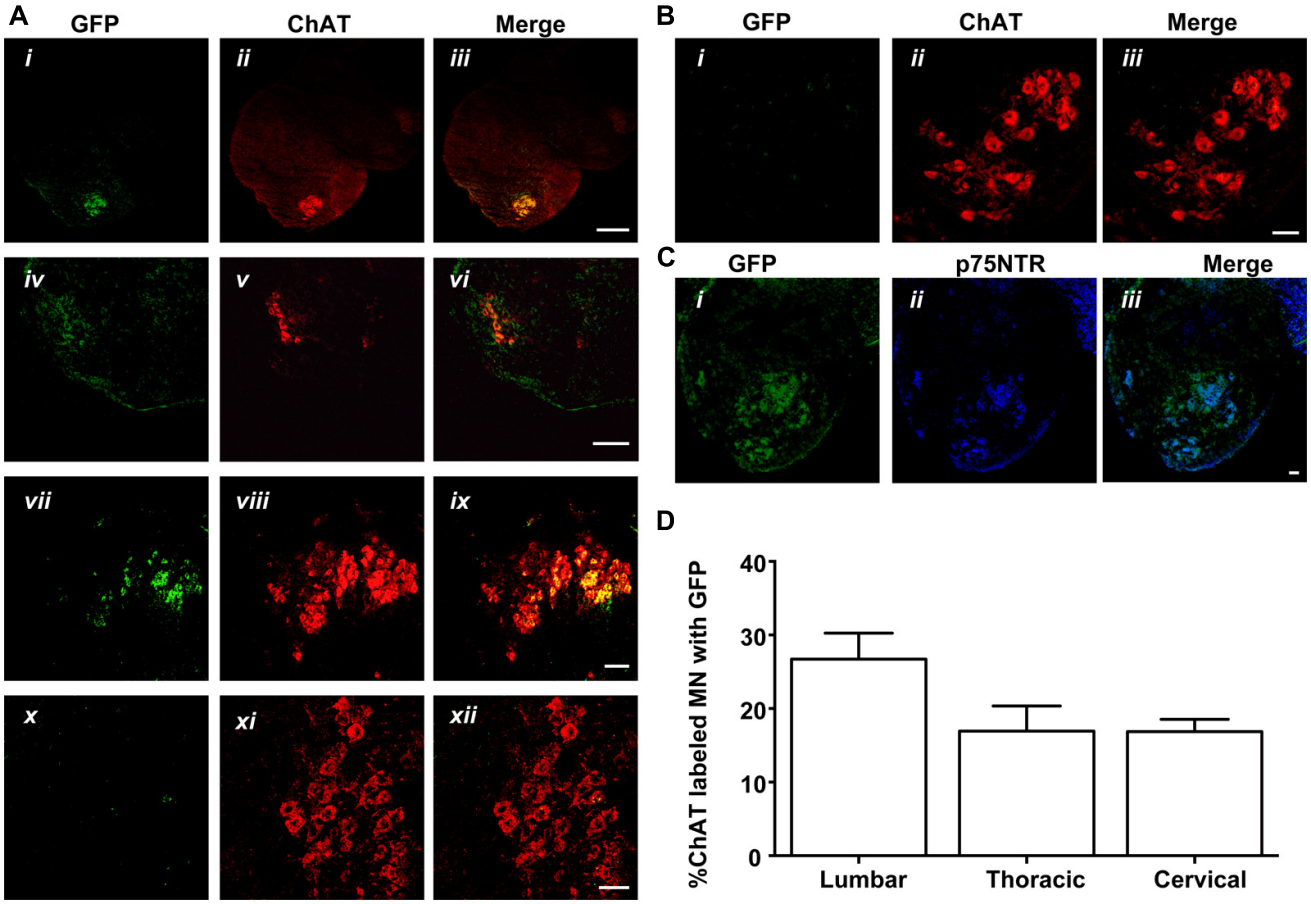
**MLR2-PEI-PEG12-pVIVO2 but not PEI-PEG12-pVIVO2 is retrogradely transported to motor neurons in neonatal mice and GFP expressed. (A)** Two doses of 75 μg of MLR2-PEI-PEG12-pVIVO2 carrying 58 μg pVIVO2 (N/P 12) was injected into neonatal mice and 72 h later spinal cords excised and examined for GFP expression (*n* = 5). Motor neurons in the lumbar (ii), thoracic (iv), and cervical (vii) regions were identified by staining with ChAT (1/5000) and GFP expression identified with chicken anti-GFP (1/500). Motor neurons that expressed GFP (i,iv,vii) always contained ChAT (iii scale bar: 100 μm; vi scale bar: 100 μm; ix scale bar: 50 μm). Motor neurons from control sections of untreated mice identified by ChAT (xi), did not contain GFP fluorescence (x) after treatment with chicken anti-GFP (1/500) scale bar: 50 μm. **(B)** Two doses of 75 μg of PEI-PEG12-pVIVO2 carrying 77.3 μg pVIVO2 (N/P 5) was injected into neonatal mice and 72 h later spinal cords excised and examined for GFP expression (*n* = 3). Lumbar sections did not contain GFP fluorescence (i) after treatment with chicken anti-GFP (1/500) and motor neurons were identified by ChAT (ii), scale bar: 50 μm. **(C)** Motor neurons expressing GFP observed in mice injected with MLR2-PEI-PEG12-pVIVO2 (i) also expressed p75NTR (1 μg/ml goat anti-p75NTR; ii) scale bar: 50 μmm. **(B,C)** M. **(D)** Percentage of lumbar, thoracic and cervical motor neurons labeled with GFP and ChAT 48 h after MLR2-PEI-PEG12-pVIVO2 given, i.p. (*n* = 5 mice with SEM).

**FIGURE 7:**
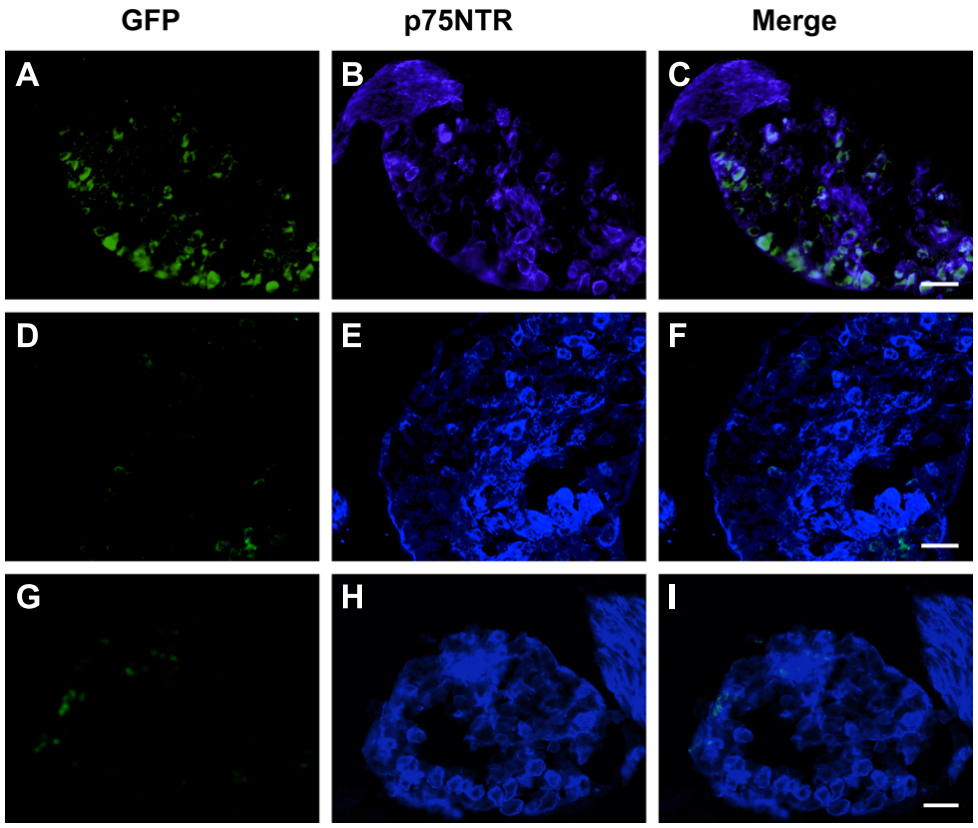
**MLR2-PEI-PEG12-pVIVO2 is retrogradely transported to dorsal root ganglia (DRG)s in neonatal mice and GFP expressed.** Two doses of 75 μg of MLR2-PEI-PEG12-pVIVO2 carrying 58 μg pVIVO2 (N/P 12) was injected into neonatal mice and 72 h L2-L4 DRGs excised and examined for GFP expression (*n* = 5). GFP expression was identified with chicken anti-GFP (1/500) and p75NTR with goat anti-p75NTR (1 μg/ml). GFP fluorescence in DRG sections was detected after treatment with chicken anti-GFP (1/500; **A**) that also contained p75NTR (**B**,**C** scale bar: 50 μm). Two doses of 75 μg of PEI-PEG12-pVIVO2 carrying 77.3 μg pVIVO2 (N/P 5) was also injected into neonatal mice and 72 h later spinal cords excised and examined for GFP expression (*n* = 3). DRG sections did not contain GFP fluorescence **(D)** in neurons that also contained p75NTR **(E,F**; scale bar: 50 μm **E,F**). Control mice non-injected DRG sections did not contain GFP **(G)** but did contain p75NTR (**H,I**; scale bar: 50 μm).

Given that MLR2 can be retrogradely delivered to the majority of motor neurons in neonatal mice, we then sought to determine the extent of gene delivery after injection of our immunogene in neonatal mice. Initially, neonatal B6 mice (average weight of 2 g) were injected with two doses of 75 μg MLR2-PEI-PEG12 carrying 58 μg of pgWiZ that expresses GFP and spinal cords examined 72 h later. However, no GFP was observed in spinal motor neurons or elsewhere (data not shown). We then used pVIVO2 that is designed specifically to enhance *in vivo* transfection through DTS and minimal CpGs. Cells transfected with pVIVO2 were identified by GFP expression. Neonatal B6 mice (*n* = 5; average weight was 2 g) were injected intraperitonealy twice with 75 μg of MLR2-PEI-PEG12 carrying 58 μg of pVIVO2, and 72 h later mice were perfused and spinal cords excised. In addition 75 μg of PEI-PEG12 carrying 77.3 μg of pVIVO2 was injected twice into three mice and 72 h later mice were perfused and spinal cords excised. Every 10th section was stained with the motor neuron marker rabbit anti-ChAT (since anti-Hb9 was not available) and chicken anti-GFP. Representative micrographs show GFP expression in lumbar, thoracic and cervical spinal cord (**Figure 6A** i,iv,vii) and motor neurons identified by ChAT (**Figure 6A** ii,v,viii). Merged images (**Figure 6A** iii,vi,ix) show that motor neurons identified by ChAT also express GFP. Sections taken from control non-injected animals had no motor neuron staining after being subject to anti-GFP (**Figure 6A** x,xi,xii), demonstrating the specificity of the anti-GFP. In addition, lumbar sections from mice injected with PEI-PEG12-pVIVO2 did not have any GFP staining (**Figure 6B** i,ii,iii). The GFP expressing neurons from mice injected with MLR2 PEI-PEG12-pVIVO2 also contained p75NTR (**Figure 6C** i,ii,iii). DRGs were also transfected with GFP (**Figure 7A**). Cells expressing GFP contained p75NTR (**Figures 7B,C**). DRGs from PEI injected animals did not contain GFP (**Figures 7D,F**) even though they expressed p75NTR (**Figure 7E**). As shown in control sections p75NTR is expressed in a high number of large diameter cells (**Figure 7H**) and as expected there was no GFP staining (**Figure 7G**). The extent of retrograde transport and gene expression was assessed for lumbar, thoracic and cervical spinal cord regions of mice injected with MLR2-PEI-PEG12-pVIVO2 by counting the number of motor neurons labeled with ChAT and GFP and with both labels. **Figure 6D** shows pooled results from six mice; 25.4 ± 2% lumbar, 18.3 ± 3.4% thoracic and 17 ± 1.7% of cervical motor neurons from the spinal cord identified by ChAT label contained GFP.

## DISCUSSION

Despite the fact that a wide range of non-viral gene delivery agents have been proposed, none have been developed that target motor neurons from the periphery. Here, we described nanoparticles that can deliver genes to motor neurons *in vivo* by an intraperitoneal route. We were able to specifically target motor neurons by including in our nanoparticle an antibody to p75NTR (MLR2) that binds and internalizes into motor neurons (Matusica et al., 2008).

The ability of p75NTR antibody MLR2 to target our nanoparticles to motor neurons from the periphery was shown by labeled MLR2 being observed in the majority of spinal motor neurons following intraperitoneal administration. MLR2 was labeled with an Atto-488 fluorophore and observed in the majority (near to 90%) of motor neurons identified by Hb9 staining, which is specific to the nucleus of developing spinal motor neurons (Arber et al., 1999). These observations indicate that MLR2 is retrogradely transported to most of the motor neurons after intraperitoneal delivery. The similar percentage of labeling across the lumbar, thoracic and cervical regions is not surprising, since motor neurons in all segments of the rodent neonatal spinal cord are known to express p75NTR (Yan and Johnson, 1988). We also observed labeled antibody in dorsal root ganglia (DRG). Previous work has shown the majority of motor neurons and sensory fibers in the spinal tract can be accessed in an identical manner by intravenous or intraperitonealy delivered agents that travel retrogradely in motor neurons and sensory fibers. Hence, intraperitoneal routes to motor neurons and dorsal root ganglia (DRG) that contain cell bodies of sensory fibers are from the circulation to terminals in the periphery. This was clearly shown by Alisky et al. (2002) where both intraperitoneal and intravenous injections of retrograde tracing agent cholera toxin subunit B (CTB) accessed all spinal motor neurons and produced identical staining. Hence, labeled MLR2 probably travels to the neuromuscular junctions via the circulation after intraperitoneal injections.

The nanoparticle comprising MLR2 conjugated to PEGylated PEI, and the GFP expressing plasmid pVIVO2 transfected motor neurons 72 h post intraperitoneal injections into 5 neonatal mice. 25.4% of lumbar, 18.3% of thoracic, and 17.0% of spinal motor neurons were transfected with pVIVO2 identified by GFP expression. When we injected PEGylated PEI carrying pVIVO2, there were no motor neurons transfected, demonstrating again that MLR2 antibody is an important component for retrograde transport to motor neurons in the spinal cord. This is also demonstrated by the fact there was no transfection in any other type of spinal cord cells when PEGylated PEI carrying pVIVO2 was injected into neonatal mice. Specificity and retrograde transport of the immunogene to motor neurons is by MLR2. Motor neurons were identified by ChAT. Since motor neurons identified by ChAT overlap with Hb9 staining in neonatal mice, this was a valid analysis (Shneider et al., 2009). The low level of transfection observed in motor neurons *in vivo* may be explained by the reported inefficiency of non-viral gene delivery (Mintzer and Simanek, 2009; Rogers and Rush, 2012). In regards to dosage, we used the same dosage of nanoparticle as we did labeled antibody (75 μg/g body weight). Hence since the same amount of antibody (when labeled) can access all the motor neurons, other areas of the nanoparticle delivery may not be optimal. Further improvements to transfection efficiency *in vivo* can be made. We already have a large payload for our nanoparticle, and although PEI is PEGylated, the whole IgG (MLR2) was not. A way to reduce interactions of the IgG with the immune system is to use the antibody binding fragments. For example, antibody fragments that lack Fc domains (FAb, Fv, scFv), have reduced interactions with the immune system and non-targeted cells through Fc receptors (Peer and Lieberman, 2011). Indeed, previous work with antibody fragments for tumor targeting using immunoliposomes carrying plasmid DNA has produced less immune reaction than whole antibodies and more sustained expression *in vivo* (Zhou et al., 2011). Hence, further improvements to transfection efficiency may be made by using FAb or scFV of MLR2 instead of the whole IgG.

This is the first report of specific gene delivery to motor neurons via the circulation. Previous viral gene delivery attempts to transfect neonatal mouse motor neurons did not have the specificity to transfect mouse motor neurons via the circulation (Towne et al., 2008). Towne et al. (2008) tested intravenous delivery of recombinant adeno-associated virus (rAAVs) expressing small hairpin RNAs targeting mutant SOD1 in the ALS mouse model. Although the AAV virus could transfect mouse motor neurons from the circulation it was not specific, it also transfected most other cell types. Towne et al. (2011) then went on to serotype their AAV viral delivery for retrograde transport and gave multiple injections to muscle groups innervated by motor neurons in neonatal SOD1 mice. Unfortunately, they could not down regulate mutant SOD1 enough to improve outcomes in ALS mice. This was suggested to be because not all motor neurons were accessed by intramuscular injections resulting in inconsistent of levels of transfection across the spinal cord. Notably, approximately 28% of lumbar, 12% of thoracic, and 18% of cervical motor neurons were transfected in neonatal mice (Towne et al., 2011). It was concluded that the lack of improvement after their viral gene therapy might be because it is difficult to access all motor neurons by intramuscular injections. In contrast, we were able to achieve motor neuron transfection after intraperitoneal injections of immunogene. We did not need to inject every muscle group to get specific transfection of motor neurons. To our knowledge we are the first group to do so. We have shown that you can transfect 25.4% of lumbar, 18.3% of thoracic, and 17.0% of spinal motor neurons after delivery of our immunogene Considering our nanoconstruct may not still be optimal, our results are hopeful for developing targeted therapy.

PEI was used to condense plasmid DNA for gene delivery *in vitro* and *in vivo*. However, PEI was modified by PEGylation to make it “stealth-like” in the circulation. Our data indicates that PEGylation reduces the toxicity branched PEI has to pure motor neurons. The viability of motor neurons *in vitro* subject to PEI conjugated to p75NTR targeting antibody MLR2 was significantly poorer than PEGylated PEI conjugated to MLR2. This result was not surprising since previous work has shown PEI without modification is toxic (Moghimi et al., 2005) causing cell stress and apoptosis (Godbey et al., 1999; Moghimi et al., 2005). Other work has shown modifying PEI by PEGylation reduces cellular toxicity (Ogris et al., 1999; Malek et al., 2009), presumably by reduction in positive charge (Merdan et al., 2003; Hoskins et al., 2012) and the formation of a hydrophilic corona around the PEI/DNA core (Merdan et al., 2005). Grafting of PEI with PEG chains thus reduces the zeta potential of PEI-based polyplexes even at high N/P ratio (Merdan et al., 2005). The zeta potential of our immunogene were negative at the N/P ratio of 12 used *in vitro* and *in vivo* (Hoskins et al., 2012). Therefore, our results showing that PEGylated PEI reduces the zeta potential and toxicity of our immunogene *in vitro* are consistent with the literature.

Although we were able to transfect motor neurons *in vivo*, our immunogene produced a low percentage (∼8%) of transfection *in vitro*. This was significantly lower than the 17–25% of motor neurons transfected throughout the spinal cord *in vivo*. This disparity between *in vitro* and *in vivo* transfection is not unusual for stable cationic constructs containing grafted stealth agents and targeting agents. For example, Höbel et al. (2011) showed that despite their relatively low *in vitro* efficacy, PEI grafted with sugars showed better *in vivo* than *in vitro* profiles and reduced toxicity. Most testing of non-viral gene delivery agents *in vitro* employs cell lines cell that rapidly divide with cell culture reagents that do not accurately mimic the *in vivo* situation (van Gaal et al., 2011). Furthermore, transfection is often undertaken without serum in the media, which does not mimic the high perecentage of serum in the circulation. Cationic polyplexes can interact with negatively charged blood components (e.g., proteins, erythrocytes), followed by the formation of aggregates. Under these conditions, precipitation can enhance the association of the delivery system with the cell surface, which can artificially elevate transfection rates with agents that are not stable in physiological media. Conversely, non-viral agents that are stable in physiological media often do not transfect efficiently in cell culture, leading to the conclusion that such systems are not worthy of further consideration. Another confounding factor with cell culture experiments is that the nuclear membrane breaks down during cell division, allowing efficient translocation of DNA into the nucleus of rapidly dividing cells that greatly facilitates transfection (Pérez-Martínez et al., 2011). We chose to test our nanoconstructs on PMN which do not divide. Our stable nanoparticle was not only PEGylated but also cross-linked to an antibody to p75NTR. Previous research has found that cross-linking amines in PEI increased the stability of PEGylated PEI and improved *in vivo* stability (Neu et al., 2007; Höbel et al., 2011). Therefore, our results producing significant *in vivo* transfection is probably reflective of the difficulty simulating *in vivo* environments *in vitro*.

PEGylation of PEI decreases the number of amines available for condensing plasmid DNA. The size of our immunogene complex at neutral charge was small (near 100 nm). This is in contrast to previous reports where branched PEI nanoconstructs complexed with DNA can be above 300 nm (Ewe et al., 2014). Conjugation to antibody MLR2 via a disulfide bridge, where amines were further reduced in the PEGylated PEI did not significantly increase the size of the complex. Previous work has shown that positively charged particles with sizes above 200 nm may be recognized and removed by the reticuloendothelial system (RES; Dash et al., 1999; Malek et al., 2009). PEGylation reduces this interaction (Ogris et al., 1999; Merdan et al., 2005; Malek et al., 2009) and also the size of the complex.

We observed no obvious off-target effects in the spinal cord and transfection of cells other than motor neurons in the spinal cord with our immunogene. Indeed, we did not observe transfection in any other cell types except for motor neurons. However, some of the p75NTR expressing cells were transfected the DRGs. p75NTR is known to be expressed in DRG cells (Yan and Johnson, 1988) and transfection of some of these cells by our immunogene again highlights the immunogene travels by receptor mediated retrograde transport to p75NTR expressing cells. The bicistronic pVIVO2 plasmid we used is specifically designed for *in vivo* transfection. The GFP reporter plasmid (pgWiZ) we used for *in vitro* transfections contains CpGs in its backbone that are known to induce immune response *in vivo* (Davies et al., 2012). pgWiZ is often used to improve humoral immune response to plasmid vaccination *in vivo* (Chamarthy et al., 2003; Grønevik et al., 2005; Rose et al., 2014). In contrast, pVIVO2 has minimal CpGs in its plasmid backbone and high levels of constitutive transgene expression has been reported for this plasmid *in vivo* (Mandke and Singh, 2012). In addition, pVIVO2 has DTS to improve nuclear entry into post mitotic cells such as motor neurons. We tried delivering pgWiZ to motor neurons by intraperitoneal injections with our immunoporter MLR2-PEI-PEG12, but found no significant expression *in vivo*. This has led us to us to conclude that plasmid design is an important component of effective non-viral gene delivery agents.

Specific delivery of genes to motor neurons is highly relevant to therapy of ALS and spinal muscular atrophy (SMA), where currently no effective therapy exists (Sreedharan and Brown, 2013). Our results show that motor neurons can be specifically transfected with peripherally administered immunogenes. Targeting motor neurons by use of the p75NTR is not surprising as this receptor is highly expressed in the embryonic period and early neonatal life (Yan and Johnson, 1988). Lentivirus that expressed heavy and light chains of rat p75NTR antibody (MC192) were recently shown transported retrogradely from the axonal tip to the cell body in an *in vitro* microfluidic culture model (Eleftheriadou et al., 2014). This is in agreement with our *in vivo* data where p75NTR antibody was found throughout the spinal cord after intraperitoneal delivery, indicating retrograde transport from terminals to ventral motor neurons throughout the spinal cord. Caution has to be taken with immunogenes use in ALS. p75NTR is down regulated in adulthood and re-expressed in injury, including ALS (Lowry et al., 2001). However, the level of p75NTR re-expression and health of the motor neurons must be sufficient for retrograde transport in the majority of motor neurons. A previous study (Copray et al., 2003) indicated 5% of L4 Lumbar motor neurons re-express p75NTR in adult ALS mice at symptomatic age. Further work is needed to determine if sufficient therapeutic genes can be delivered in adult ALS animal models via p75NTR targeting immunogenes. Since SMA is a disease often occuring in childhood (Arnold and Burghes, 2013), p75NTR targeting immunogenes could be trialed in SMA mice.

## CONCLUSION

Our current research demonstrates the suitability of p75NTR targeting immunogenes to transfect motor neurons from the periphery in neonatal mice, but further work is required for use in adult animals.

## Conflict of Interest Statement

Author Robert A. Rush is now retired from Flinders University and holds the position of Managing Director at Biosensis Pty. Ltd. which has commercial interest involving the MLR2 antibody used in this study. No other authors have conflicting interest.
